# Cell Progression and Survival Functions of Enzymes Secreted in Extracellular Vesicles Associated with Breast and Prostate Cancers

**DOI:** 10.3390/cells14070468

**Published:** 2025-03-21

**Authors:** Cosmos Ifeanyi Onyiba, Niwasini Krishna Kumar, Christopher J. Scarlett, Judith Weidenhofer

**Affiliations:** 1 School of Biomedical Sciences and Pharmacy, College of Health, Medicine and Wellbeing, University of Newcastle, Ourimbah, NSW 2258, Australia; 2 Hunter Medical Research Institute, New Lambton Heights, NSW 2305, Australia; 3 School of Health Sciences, International Medical University, Kuala Lumpur 57000, Malaysia; 4 School of Environmental and Life Sciences, College of Engineering, Science and Environment, University of Newcastle, Ourimbah, NSW 2258, Australia

**Keywords:** extracellular, vesicles, proteins, enzymes, breast, prostate, cancer, tumor, progression, survival

## Abstract

Extracellular vesicles (EVs) are membrane-bound cargoes secreted by normal and pathological cells. Through their protein, nucleic acid, and lipid cargoes, EVs mediate several cellular processes, such as cell–cell communication, cell development, immune response, and tissue repair. Most importantly, through their enzyme cargo, EVs mediate pathophysiological processes, including the pathogenesis of cancer. In this review, we enumerate several enzymes secreted in EVs (EV enzyme cargo) from cells and patient clinical samples of breast and prostate cancers and detail their contributions to the progression and survival of both cancers. Findings in this review reveal that the EV enzyme cargo could exert cell progression functions via adhesion, proliferation, migration, invasion, and metastasis. The EV enzyme cargo might also influence cell survival functions of chemoresistance, radioresistance, angiogenesis, cell death inhibition, cell colony formation, and immune evasion. While the current literature provides evidence of the possible contributions of the EV enzyme cargo to the progression and survival mechanisms of breast and prostate cancers, future studies are required to validate that these effects are modified by EVs and provide insights into the clinical applications of the EV enzyme cargo in breast and prostate cancer.

## 1. Introduction

Enzymes are proteins that catalyze various biological reactions in living organisms [1]. Given their ubiquitous nature, enzymes are involved in both physiological and pathological processes [2,3], including the pathogenesis of various cancers, such as breast and prostate cancer [4,5]. According to recent epidemiological data from the United States, breast and prostate cancers are the most frequently diagnosed cancers among women and men, respectively [6], and the world [7]. In Australia, breast and prostate cancers are the second and first most frequently diagnosed cancers, respectively [8]. Given these statistics, breast and prostate cancers seem to have similar trends in terms of their high frequencies among women and men globally. Moreover, while the breast and prostate serve different purposes, malignancies that develop in both tissues are similar in that they (1) depend on hormones [9,10], (2) portray similar genetic underpinnings [11,12], (3) frequently exhibit large proportions of indolent disease [13], and (4) respond to similar therapy, such as endocrine therapy [14]. These similarities justify the simultaneous focus of this review on breast and prostate cancers.

Extracellular vesicles (EVs) are 30–1000 nm in diameter cargo deliverers secreted by almost every cell in the body in both the normal and pathologic state [15]. Although there are continuously evolving classifications, EVs are broadly classified according to their size and biogenesis into ectosomes (100–1000 nm) and exosomes (30–150 nm) [16]. However, the usage of the terms “exosomes” and “ectosomes” has recently been discouraged by the International Society for Extracellular Vesicles (ISEV) due to insufficient evidence to support their biogenesis [16]. Therefore, the term “EV” has been established by ISEV as the adopted nomenclature for both exosomes and ectosomes. According to ISEV 2023 guidelines, EVs are now characterized based on size (small EVs: <200 nm and large EVs: >200 nm), density (low, medium, and high), and biochemistry (protein markers) [16].

**Figure 1:**
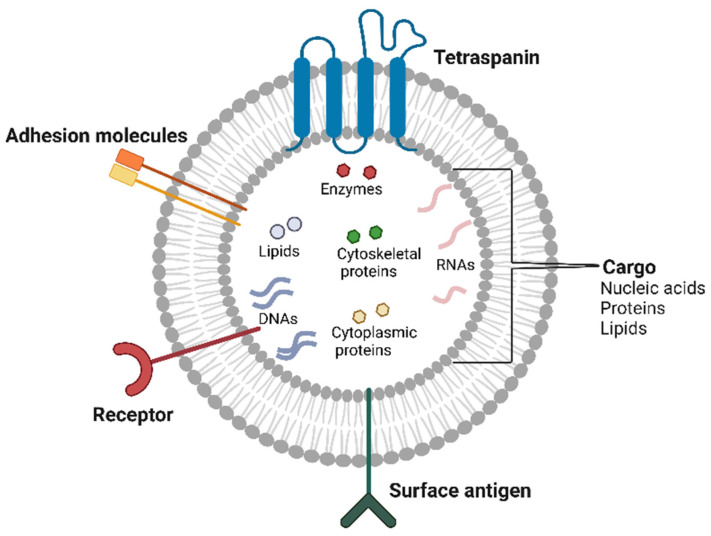
Components of extracellular vesicles. EVs are membrane-bound carriers of nucleic acids, proteins, and lipids. EVs are structurally composed of specific proteins, including receptors, surface antigens, adhesion molecules, and tetraspanins. The image was created with BioRender.com (accessed on 14 January 2025).

EVs carry cargoes of a wide range of important bioactive molecules, including lipids, proteins (such as cytoskeletal proteins, enzymes, cytoplasmic proteins, and RNA-binding proteins), RNAs (mRNAs, tRNAs, miRNAs, DNAs, and other RNAs), and DNAs (such as mtDNA, dsDNA, and ssDNA) [17] (Figure 1).

Given their ability to transfer molecules that influence gene expression, EVs play vital roles in cell–cell communication, cell development, immune response, and tissue repair [18]. Additionally, several studies have reported certain differences in EVs of certain pathological cells compared to their corresponding normal cells [18]. These differences may include higher secretion rates of EVs, alterations in the expression of EV structural proteins, and functional differences in specific EV content (e.g., proteins and nucleic acids). Since cancers remodel their microenvironment by transferring various molecules (including enzymes) between their cells via secretion and uptake of EVs [19], there is a need to investigate the contributions of these molecules to the pathogenesis of cancers. This would provide a basis for exploring their diagnostic, prognostic, and therapeutic relevance to various cancers, including breast and prostate cancers. Therefore, in this review, we provide an overview of enzymes in the pathogenesis of breast and prostate cancer. Most importantly, we discuss the contributions of enzymes secreted in EVs associated with breast and prostate cancers to the progression and survival of both cancers.

## 2. Roles of Enzymes in the Pathogenesis of Breast and Prostate Cancers

**Figure 2:**
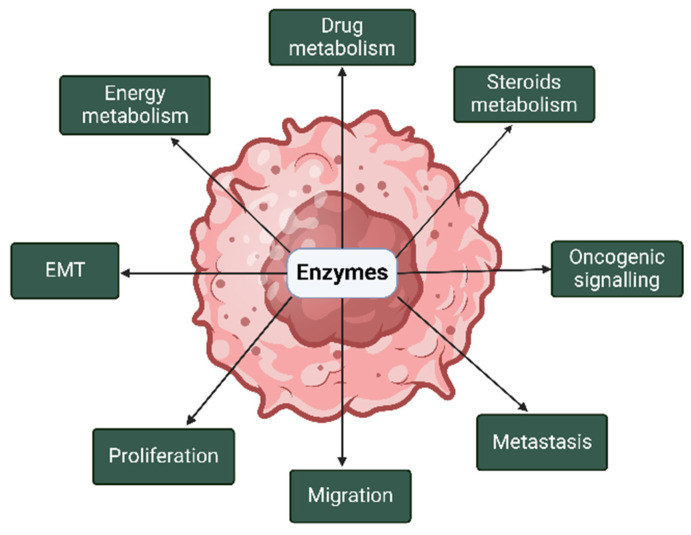
Roles of enzymes in the pathogenesis of breast and prostate cancers. In breast and prostate cancers, enzymes are implicated in metabolism, oncogenic signaling, proliferation, and epithelial–mesenchymal transition (EMT), among others. The image was created with BioRender.com (accessed on 14 January 2025).

Enzymes have been implicated in the pathogenesis of breast and prostate cancers through their contribution to various malignant processes in both cancers (Figure 2).

In breast cancer, there is a marked increase in the activities of glycolytic enzymes, such as pyruvate kinase (PK), hexokinase (HK), enolase, phosphofructokinase (PFK), and aldolase, as compared to normal breast tissues [20]. Similarly, in prostate cancer, there is an increase in the activities of PK, HK, and PFK as compared to normal prostate tissues [21]. This indicates a pivotal role for these glycolytic enzymes in the supply of energy to drive the progression of breast and prostate cancers, as explained by the Warburg effect [22,23], which could be harnessed as a treatment strategy for both cancers [24,25,26].

In the metabolism of drugs and xenobiotics in breast and prostate cancers, several enzymes have been implicated. In breast cancer, there is an increased expression of UDP-glucuronosyltransferases [27] and various cytochrome P450 (CYP) enzymes, such as CYP2A6 and CYP1B1, as compared to normal breast tissues [28]. Similarly, the upregulated expression of various CYP enzymes, such as CYP27A1 and CYP2R1, in prostate cancer has been documented [29]. The expression pattern of these drugs and xenobiotics metabolizing enzymes offer opportunities for the development of personalized treatments for both breast and prostate cancers [28,29]. Given that breast and prostate cancers are both driven by sex steroid hormones [9], the involvement of sex steroid metabolizing enzymes, such as 17β-hydroxysteroid dehydrogenases (17βHSDs), in the pathogenesis of both cancers has been documented [30,31,32,33,34,35,36]. The 17βHSD family of enzymes catalyzes the conversion (oxidation/reduction) of hydroxyl groups in the 17β position of the backbones of steroids [37]. This conversion by 17βHSDs can lead to various outcomes, including the production of the active form of estrogen and androgen (estradiol and testosterone, respectively), which drive breast and prostate cancers, respectively [38]. Moreover, 17βHSD types 1 and 2 have been shown to predict patient outcomes for breast cancer [39,40], whereas 17βHSD type 3 has been shown to offer therapeutic opportunities for prostate cancer treatment [41].

In the progression of breast and prostate cancers, several enzymes have been found to act as either tumor suppressors or tumor promoters. A typical example of such enzymes is the silent information regulator 2 (sir2) family of proteins (sirtuins). Sirtuins are classified as class III histone deacetylases that transfer an acetyl group to the ribose moiety of adenosine diphosphate (ADP), producing 2-O-acetyl-ADP-ribose (o-AADPR) and NAM, a feedback inhibitor of sirtuins [42]. Seven sirtuins (SIRT1–7) have been identified in mammals [43], which function as NAD-dependent deacetylases (SIRT1–3 and SIRT5–7) and ADP-ribosyl transferases (SIRT4 and 6) [44]. In our recent review [44], we extensively revealed that sirtuins modulate various molecular targets to mediate tumor-suppressing and/or tumor-promoting effects, thus offering therapeutic opportunities against both cancers. Furthermore, cystathionine β-synthase and cystathionine γ-lyase are hydrogen sulfide (H2S)-producing enzymes whose constitutive upregulation in breast and prostate cancers [45,46] has been shown to promote the aggressiveness of breast and prostate cancers through increased production of H2S [47,48]. H2S has been shown to significantly drive several oncogenic signaling pathways, including the JAK/STAT, Ras/Raf/MEK/ERK, and PI3K/AKT/mTOR signaling pathways [47].

Another class of enzymes implicated in the aggressiveness of breast and prostate cancers is the hyaluronan synthases (HASs), which comprise three isoforms (HAS1–3) that differ by their expression patterns, enzymatic activities, and subcellular locations [49,50]. HASs are responsible for the biosynthesis of hyaluronan (HA), an extracellular matrix molecule composed of a large glycosaminoglycan polymer. Studies have reported an increased expression of HASs and HA in breast and prostate cancers, which suggests the potential diagnostic and prognostic relevance of HASs in both cancers [51,52,53,54]. As a mechanism, HA promotes the aggressiveness of breast and prostate cancers via the induction of epithelial–mesenchymal transition (EMT) to drive migration and metastasis of both cancers [53,55]. Moreover, EMT has been found to confer migratory capacity, radio/chemoresistance, invasiveness, and resistance to apoptotic cell death in cancers [56]. Based on these findings, HASs offer opportunities for the identification of potential drug targets for the treatment of breast and prostate cancers [57].

Additionally, the role of proteases in breast and prostate cancers has been extensively investigated [58,59,60,61,62]. For instance, matrix metalloproteinases (MMPs) have been found to promote proliferation and metastasis of both breast and prostate cancers [63,64,65,66]. Also, protein kinases have been found to play pivotal roles in breast and prostate cancers [67,68,69,70]. A typical example is protein kinase D, which has been shown to promote metastasis of breast and prostate cancers [71,72,73]. Overall, enzymes are indicated in the pathogenesis of breast and prostate cancers. Therefore, in this review, we explore the cell progression and survival functions of enzymes secreted in EVs associated with breast and prostate cancers.

## 3. Cell Progression and Survival Functions of Enzymes Secreted in EVs Associated with Breast and Prostate Cancers

Perhaps the main relevance of metabolic reprogramming in cancers is embodied by “reprogrammed enzymes” that exhibit malignant functions [74,75,76,77] in addition to their canonical functions as biological catalysts. This enzyme reprogramming may involve the selective upregulation or downregulation of enzymes either via alteration in gene expression [78] or suggestively via uptake of EVs containing enzymes and/or their modulators from neighboring cells. Moreover, the selective inclusion of enzymes in EVs may serve to transport certain enzymes between cells, which can influence pathophysiological processes, including the pathogenesis of cancer [79]. A crucial example is the discovery that EVs from cancer cells can selectively include purinergic enzymes (e.g., ectonucleotidases) in their cargo to influence cancer progression [80].

**Figure 3:**
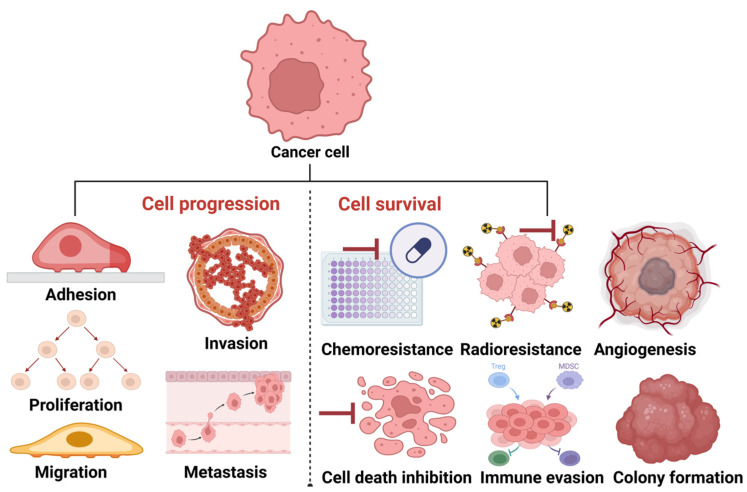
Progression and survival signals in cancer. To progress, cancer cells turn on tumor progression signals, such as adhesion, proliferation, migration, invasion, and metastasis. To survive, cancer cells turn on tumor adaptative signals, such as chemo/radioresistance, angiogenesis, colony formation, and cell death inhibition. The image was created with BioRender.com (accessed on 20 March 2025).

Although cancer progression has been previously described as a combination of genetic and epigenetic signals that confer a growth advantage to cancers [81], it is also described as a learning process that ensures the survival of cancers [82]. Based on the above descriptions, it is apparent that the pathogenesis of cancer involves the synergistic function of two broad, mutually connected signals: cell survival and cell progression (Figure 3).

Since tumor functions, such as adhesion, proliferation, migration, invasion, and metastasis, are mechanisms supporting cancer cell progression [83], we opine that tumor adaptative functions, such as chemoresistance, radioresistance, angiogenesis, cell death inhibition, colony formation, and immune suppression (evasion), support cancer cell survival. Therefore, this review explores the contributions of enzymes secreted in EVs to these cancer cell progression and survival mechanisms.

Using protein-based and/or mRNA-based methods, studies included in this review have identified and quantified enzymes in EVs from breast/prostate cancer cells and clinical samples (plasma and urine) from patients with breast/prostate cancer. However, most of these studies are faced with limitations of non-uniformity in the methods of isolating EVs, and the lack of enzyme activity assays for verifying the active form of identified enzymes. Barring these limitations, we enumerate several enzymes detected in EVs associated with breast and prostate cancers and discuss their malignant functions in terms of their contributions to the progression and survival of both cancers (summarized in Table 1 for breast cancer and Table 2 for prostate cancer).

### 3.1. Enzymes Secreted in EVs Associated with Both Breast and Prostate Cancers

#### 3.1.1. Adenosine Triphosphate (ATP) Citrate Lyase (ACLY)

ACLY has been identified in EVs associated with cancers of the breast [84] and prostate [99]. ACLY expression is upregulated in both breast [110] and prostate [111,112] cancers. ACLY has been reported as a cell survival factor in both breast and prostate cancers. For instance, silencing of ACLY by siRNA significantly decreased cell viability and increased apoptosis of MCF-7 breast cancer cells [110]. Consistent with this finding, Velez et al. demonstrated that ACLY inhibition by a combination of bempedoic acid (BA) and palbociclib decreases the viability of MDA-MB-231 breast cancer cells and that ACLY inhibition by BA induces apoptosis in MDA-MB-231 breast cancer cells via elevation of the apoptotic markers cPARP and p-c-Jun [113]. Moreover, the inhibition of ACLY has also been found to induce apoptotic cell death in other cancers [114]. On the other hand, Gao et al. showed that pharmacologic inhibition of ACLY by hydroxycitrate tribasic induces apoptosis of PC-3 and LNCaP prostate cancer cells [115]. The authors further demonstrated that ACLY inhibition by siRNAs enhances the apoptosis of PC-3 and LNCaP prostate cancer cells induced by Cucurbitacin B [115] and that Cucurbitacin B downregulates the expression of ACLY in PC-3 and LNCaP prostate cancer cells [115]. The inhibition of apoptosis by ACLY is at least in part due to the inhibition of ROS, as shown by Migita et al. through siRNA inhibition of ACLY in LNCaP prostate cancer cells [116]. Moreover, it has been previously demonstrated in earlier studies that ACLY knockdown by siRNAs inhibits cell cycle progression and induces apoptosis of PC-3M prostate cancer cells [117]. Substantiating these findings, the tumor suppressor miR-22 [118] was found to mediate its anticancer effect by inhibiting ACLY activity in PC-3 prostate cancer cells [112]. Together, these findings indicate that ACLY contributes to the survival of breast and prostate cancers via cell death inhibition. However, it would be interesting to investigate the contributions of ACLY to the progression of both cancers.

#### 3.1.2. Enolase (ENO)

Among the ENO isoforms, ENO1 and ENO2 have been identified in EVs associated with cancers of the breast [85,86,87] and prostate [99]. Consistent with this finding, upregulated expressions of ENO1 and ENO2 have been reported in both breast [119,120] and prostate [121,122] cancers. There is limited information regarding the relevance of ENO to the progression and survival of breast and prostate cancers. Notwithstanding, Tu et al. [123] established that induced expression of ENO1 by 4-hydroxy-tamoxifen (4-OHT) confers 4-OHT drug-induced resistance in MC7 breast cancer cells via induction of the ER/NFkB pathway [123]. Chen et al. showed that the ENO1 inhibitor HuL227 downregulates tumor growth, angiogenesis, and recruitment of CCR2+ inflammatory monocytes in the PC-3 subcutaneous xenograft model of prostate cancer [124]. This suggests that ENO1 contributes to the survival of breast cancer via chemoresistance while contributing to the survival of prostate cancer via angiogenesis and immune evasion. The contributions of ENO2 to the survival of breast and prostate cancers need to be investigated.

In another study, inhibition of ENO1 by HuL227 halted inflammation-induced migration of PC-3 prostate cancer cells and reduced CCL2 or TGFβ-enhanced migration of PC-3 and DU145 prostate cancer cells [124]. This indicates a role for ENO1 in the progression of prostate cancer via the promotion of migration. Studies are required to determine the contributions of ENO1 to the progression of breast cancer. Although there is a paucity of data on the relevance of ENO2 to the progression of breast and prostate cancers, ENO2 mRNA was found to be elevated in breast cancer lymph node metastases compared to primary breast tumors [125], which may suggest its role in breast cancer progression. Similarly, ENO2 expression was also upregulated in the estrogen receptor-positive subset of 36 invasive ductal breast carcinomas compared to non-tumor counterparts [126]. Also, Zhou et al. opined in their systematic review of five studies that ENO2 contributes to the metastasis of prostate cancer [122]. Given the limited information, more studies are required to investigate the malignant functions of ENO1 and ENO2 in both cancers before a conclusion can be reached.

#### 3.1.3. Fatty Acid Synthase (FASN)

FASN has been identified in EVs associated with breast [87] and prostate [99] cancers. It is also highly expressed in cancers of the breast [127,128,129] and prostate [130,131]. In the study by Menendez et al., FASN inhibition by the mycotoxin cerulenin sensitizes SK-BR-3 breast cancer cells to the cytotoxic effect of the anticancer drug docetaxel via downregulation of HER-2/neu oncogene expression [127]. Also, FASN inhibition by either siRNA or exogenous inhibitor increases cisplatin-induced apoptotic cell death in MDA-MB-231 breast cancer cells [128]. To corroborate this finding, Schroeder et al. demonstrated that FASN inhibition by the FASN inhibitor C75 induces apoptotic cell death in BT-474, MCF-7, MDA-MB-231 breast cancer cells by promoting ROS-dependent mitochondrial cytochrome c release and upregulating pro-apoptotic factors, such as BIM, NOXA, and PUMA [129]. In an earlier study by Jin et al., FASN inhibition by lapatinib or C75 was found to inhibit the invasion of SK-BR-3 and BT-474 breast cancer cells [123]. Using LNCaP-LN3 prostate cancer cells, Oh et al. demonstrated that FASN inhibition by FAS inhibitors (TVB-3166, GSK2194069, and Fasnall) results in metabolic alterations in [132], which may negatively affect the cancer’s metabolic reprogramming and survival. Additionally, FASN inhibition by triclosan or siRNAs induced apoptosis of LNCaP prostate cancer cells [133,134]. Moreover, studies have demonstrated that FASN inhibition by C75 sensitizes PC-3 and LNCaP prostate cancer cells to the apoptotic and growth-inhibiting effects of radiotherapy [135,136]. Corroborating these findings, Huang et al. reported a downregulated expression of FASN in patients with prostate cancer who were treated with androgen deprivation therapy and chemotherapy [137], thus suggesting FASN as a molecular target against prostate cancer. Together, the above findings indicate that FASN contributes to the survival of breast and prostate cancers via chemoresistance and cell death inhibition.

Furthermore, Li et al. demonstrated that FASN inhibition by cerulenin results in decreased migration of MCF-7-MEK5 breast cancer cells [138]. Corroborating this finding, Xu et al. demonstrated that FASN inhibition by cerulenin decreases the migration of SK-BR-3 breast cancer cells [125]. Also, upregulated FASN expression and activities were detected in brain metastasis of breast cancer, which was evidenced by the detection of FASN mRNA and lipid products, such as monosaturated fatty acids (e.g., triacylglycerols), in brain metastasis of BT-474 breast cancer cells in mice [126]. Moreover, a recent study demonstrated that FASN inhibition by TVB-2640 slows migration and reduces invasion of MDA-MB-231 breast cancer cells and its brain metastatic variant MDA-MB-231BR [139]. Using LNCaP and C4-2 prostate cancer cells, Huang et al. showed that FASN inhibition by siRNAs and cerulenin decreased the proliferation of prostate cancer [137]. Also, Yoshii et al. demonstrated that FASN inhibition by siRNAs reduces the proliferation and migration of LNCaP prostate cancer cells [140]. A recent study by De Piano et al. also demonstrated that FASN inhibition by siRNAs results in decreased migration of 1542 and PC-3 prostate cancer cells [141]. Together, the above studies indicate that FASN contributes to the progression of breast cancer via the promotion of migration, invasion, and metastasis while contributing to the progression of prostate cancer via the promotion of proliferation and migration.

#### 3.1.4. Focal Adhesion Kinase (FAK)

FAK has been identified in EVs associated with cancers of the breast [88] and prostate [100]. Consistent with this, upregulated FAK expression has been reported in both breast [142] and prostate [143] cancers. In a study by Li-Hui et al., it was demonstrated that the downregulation of FAK results in the loss of cellular adhesion and induces the apoptosis of BT474 breast cancer cells [144]. Corroborating this finding, Vita et al. demonstrated that inhibition of FAK downregulates the AKT and ERK1/2 survival pathways and induces the apoptosis of BT-20 breast cancer cells [145]. Another earlier study by Satoh et al. demonstrated that FAK inhibition by antisense oligonucleotides sensitizes ZR-75-1, MDA-MB-231, and MCF-7 breast cancer cells to the cytotoxic effects of camptothecin [146], a plant-derived anticancer agent [147]. Also, the inhibition of FAK by the FAK inhibitor PF87 was reported to sensitize BT-474 and MDA-361 breast cancer cells to the cytotoxic effects of the anti-Her2+ breast cancer agent trastuzumab [148]. In mice infected with PC-3 prostate cancer cells, FAK inhibition by shRNA prevented the growth of prostate tumors via induction of apoptosis [149]. Corroborating this finding, another study demonstrated that FAK inhibition by co-administration of defactinib and docetaxel reduces the viability of docetaxel-sensitive and resistant PC-3 and DU145 prostate cancer cells, inhibits the growth of PC-3 xenografts, and induces apoptosis in patient-derived prostate tumor explants [150]. Moreover, Johnson et al. demonstrated that overexpression of FAK promotes cell survival of PC-3 prostate cancer cells via an increase of clonogenic activity (colony formation) [151]. Together, these studies indicate that FAK contributes to the survival of breast and prostate cancers via chemoresistance and cell death inhibition.

FAK inhibition by PF878 was shown to suppress migration but not the proliferation of BT-474 and MDA-361 breast cancer cells [148]. Similarly, inhibition of FAK was also shown to reduce cell–matrix attachment (cell adhesion) and migration of endocrine-resistant variants of MCF-7 breast cancer cells [152]. Also, Sumitomo et al. demonstrated that migration of LNCaP prostate cancer cells was halted following the inhibition of FAK by neutral endopeptidase (NEP) [153]. NEP is a surface enzyme of prostatic epithelial cells that inactivates neuropeptides involved in androgen-independent prostate cancer progression [154]. Consistent with this, Lacoste et al. demonstrated that bombesin, a substrate of NEP, induces motility of PC-3 prostate cancer cells via activation (tyrosine phosphorylation) of FAK [155]. Moreover, phosphorylation of FAK at tyrosine 861 residue has been reported to enhance the migration of PC-3 prostate cancer cells [156]. Additionally, several proteins (such as S100A9 and RAB11A) implicated in the proliferation, invasion, migration, and metastasis of PC-3, VCaP, and DU145 prostate cancer cells have been found to mediate their malignant functions via FAK [157,158]. Overall, the above studies indicate that FAK contributes to the progression of breast cancer via the promotion of adhesion and migration while contributing to the progression of prostate cancer via the promotion of proliferation, invasion, migration, and metastasis.

#### 3.1.5. Pyruvate Kinase (PK)

Among the PK isoforms, PKM2 has been identified in EVs associated with breast [86,87] and prostate [99,101] cancers. Also, PKM2 is highly expressed in breast [159,160] and prostate [161,162] cancers. Overexpression of PKM2 has been associated with chemosensitivity to epirubicin and 5-fluorouracil in patients with breast cancer [163]. In contrast, there is evidence that chemotherapeutic drugs, including TAM and lapatinib, exert their cytotoxic effects against breast cancer by inhibiting PKM2 [164,165,166]. Additionally, a combination of PKM2 inhibitors (gliotoxin and shikonin) was found to sensitize high-density MDA-MB-231 breast cancer cells to the cytotoxic effect of vincristine, which was substantiated by increased apoptosis of the breast cancer cells [167]. Also, Dey et al. demonstrated that PKM2 knockdown by siRNAs induces autophagic cell death of DU145 prostate cancer cells via inhibition of the Akt/mTOR signaling [161]. The authors further demonstrated that PKM2 knockdown by siRNAs alters metabolism and inhibits viability and colony formation of DU145 prostate cancer cells [161]. Moreover, it has been previously noted that silencing of PKM2 by siRNAs decreases colony formation of PC-3 and LNCaP prostate cancer cells [168]. Corroborating these findings, a recent study by Jiang et al. revealed that PKM2 knockdown by a novel PKM2 inhibitor, compound 3h, induces apoptotic and autophagic cell death of LNCaP prostate cancer cells [169]. Together, these studies indicate that PKM2 contributes to the survival of breast cancer via chemoresistance and cell death inhibition while contributing to the survival of prostate cancer via cell death inhibition and colony formation.

Furthermore, the Knockdown of PKM2 by specific siRNAs was reported to suppress growth and migration as well as induce G2/M phase cell cycle arrest of MDA-MB-231 and HCC1937 breast cancer cells [159]. In another study, the knockdown of PKM2 by siRNA suppressed EMT and consequently downregulated migration and invasion of MDA-MB-231 and MCF-7 breast cancer cells [170]. Also, Guo et al. demonstrated that PKM2 silencing by siRNAs inhibits migration, invasion, and EMT of DU145 and PC-3 prostate cancer cells [162]. As a mechanism, the authors further established that PKM2 overexpression promotes migration, invasion, and metastasis of DU145 and PC-3 prostate cancer cells via the ERK1/2/c-Jun/Cox-2 signaling pathway [162]. Together, these findings suggest that PKM2 contributes to the progression of breast cancer via the promotion of migration and invasion while contributing to the progression of prostate cancer via the promotion of migration, invasion, and metastasis.

### 3.2. Enzymes Secreted in EVs Associated with Breast Cancer (with No Current Evidence in Prostate Cancer)

#### 3.2.1. Phosphoglycerate Kinase 1 (PGK1)

While PGK1 mRNA is identified in EVs associated with breast cancer [86], it is overexpressed in breast cancer [171,172]. There is limited information on the relevance of PGK1 to breast cancer cell survival and progression. Notwithstanding, a functional study by Deyuan et al. demonstrated that PGK1 knockdown by lentivirus-mediated transfection inhibits invasion and reverses EMT of MDA-MB-231 and MCF-7 breast cancer cells [173], thus suggesting PGK1 is a cell progression factor in breast cancer. Moreover, an earlier study by Dan et al. demonstrated that 17β-HSD5 knockdown-induced overexpression of PGK1 inhibits apoptosis and promotes cell viability and proliferation of MCF-7 breast cancer cells [174], indicating PGK1 as a cell survival and progression factor in breast cancer. In addition, correlational studies involving patients with breast cancer revealed an association of PGK1 with paclitaxel resistance [175] and cell survival factors, such as HIF-1α [173] and immune checkpoints [176], although these need to be further investigated in future studies.

#### 3.2.2. Phosphoglycerate Mutase 1 (PGAM1)

Consistent with the expression of PGAM1 mRNA in EVs associated with breast cancer [86], there is an upregulated expression of PGAM1 in breast cancer [177,178,179]. In a study by Zhang et al., it was reported that the knockdown of PGAM1 in 4T1, MCF-7, and MDA-MB-231 breast cancer cells resulted in a marked reduction in M2 polarization, migration, and interleukin-10 (IL-10) production of macrophages [179]. M2 macrophages have been reported to act as immunoregulators by suppressing inflammatory responses via the production of anti-inflammatory cytokines, such as IL-10 [180]. Corroborating the above findings, another study by Zhang et al. revealed that PGAM1 suppression in triple-negative breast cancer (TNBC) synergizes with anti–PD–1 immunotherapy to diminish breast cancer cell survival [178]. Zhang et al. also demonstrated that the depletion of PGAM1 by shRNA reduces the migration of MDA-MB-231 breast cancer cells [181]. Supporting this finding, a recent study demonstrated that the depletion of PGAM1 by siRNA inhibited the proliferation, invasion, migration, and EMT of MDA-MB-231 and MCF-7 breast cancer cells [177]. Overall, these findings indicate that PGAM1 is a cell survival factor via immune cell suppression and a cell progression factor via the promotion of proliferation, invasion, migration, and EMT in breast cancer.

#### 3.2.3. Glucose-6-Phosphate Dehydrogenase (G6PDH)

G6PDH has been identified in EVs associated with breast cancer [87]. It is also consistently overexpressed in breast cancer [182,183]. Using MCF-7 breast cancer cells, Luigi et al. demonstrated that G6PDH overexpression reduces autophagic cell death, as evidenced by the decreased expression of autophagosome formation markers, such as LAMP1, p62, and LC-3 [182]. The authors further demonstrated that G6PDH overexpression induces MCF-7 breast cancer cell resistance to lapatinib [182], a tyrosine kinase inhibitor used in the treatment of patients with breast cancer [184]. Consistent with these findings, using MCF-7 and MDA-MB-231 breast cancer cells, Yin et al. demonstrated that G6PDH inhibition by 6-aminonicotinamide caused a marked increase in the production of reactive oxygen species (ROS), inducing autophagic cell death [183]. These findings suggest that G6PDH promotes breast cancer cell survival via cell death inhibition and chemoresistance [185]. The role of G6PDH in breast cancer cell progression is yet to be investigated.

#### 3.2.4. Glyceraldehyde 3-Phosphate Dehydrogenase (GAPDH)

GAPDH has been identified in EVs associated with breast cancer [86,87,89]. Consistent with its expression in the EVs, GAPDH is also overexpressed in breast cancer [186]. Although there is a paucity of information regarding the relevance of GAPDH to breast cancer cell survival, studies have established that the enzyme is largely involved in the adaptation of breast cancer to hypoxia. For instance, Yasuki et al. demonstrated that hypoxia induces the upregulation of GAPDH in MCF-7 breast cancer cells and that this hypoxia-induced expression of GAPDH is mediated by hypoxia-inducible factor (HIF)-1α. HIF-1α is a master regulator of how cancer cells respond to hypoxia and is crucial for cancer cell survival as it activates the transcription of genes involved in resistance to radiation therapy, chemotherapy, and angiogenesis [187]. Moreover, angiogenesis is important for the supply of oxygen and nutrients to tumors for their continuous growth and survival [145]. Therefore, given that GAPDH expression is upregulated by HIF-1α, future studies are required to investigate the relevance of the HIF-1α/GAPDH axis to breast cancer cell survival in terms of radioresistance, chemoresistance, and angiogenesis. In addition, future studies are needed to determine the relevance of GAPDH in the proliferation, migration, invasion, and metastasis of breast cancer cells since GAPDH expression is found to be associated with breast cancer cell proliferation and tumor aggression in patients with breast cancer [186].

#### 3.2.5. A Disintegrin and Metalloproteinases (ADAM) 9 and 10

Among the ADAM isoforms, ADAM9 and ADAM10 have been identified in EVs associated with breast cancer [90,91,92]. Consistent with this, ADAM9 and ADAM10 are overexpressed in breast cancer [188,189,190,191]. Silencing ADAM9 by siRNA inhibited the migration of BT-549 breast cancer cells [192] and the invasion of MDA-MB-231 breast cancer cells [193]. Corroborating this finding, a recent study by Song et al. demonstrated that the depletion of ADAM9 expression by shRNA inhibits the proliferation, migration, and invasion of MCF-7 and MDA-MB-231 breast cancer cells [189]. The authors further demonstrated that depletion of ADAM9 impedes growth and increases radiosensitivity and apoptosis of MCF-7 and MDA-MB-231 breast cancer cells [189]. Moreover, a mechanistic study revealed that ADAM9 promotes the progression of MDA-MB-231 breast cancer cells by activating the AKT/NF-κB pathway [188], an oncogenic signaling pathway implicated in many cancers [194]. Together, these findings establish that ADAM9 contributes to breast cancer cell survival via cell death inhibition and radioresistance while contributing to breast cancer cell progression via the promotion of proliferation, migration, and invasion.

Inhibition of ADAM10 by INCB8765 was reported to improve trastuzumab response against BT-474 and SK-BR-3 breast cancer cells [195]. Also, ADAM10 knockdown by siRNA in MDA-MB-231 breast cancer cells induces apoptosis and reduces the IC50 value of the anticancer drugs paclitaxel and adriamycin (ADR) [190]. In further analysis, it was demonstrated that ADAM10 inhibits apoptosis and confers chemoresistance in MDA-MB-231 breast cancer cells via upregulation of CD44 and PrPc proteins [190]. CD44 promotes chemoresistance in cancers [196], while PrPc protects cells against oxidative stress [197]. Moreover, low-level oxidative stress activates cell survival signals, while high levels induce apoptotic cell death [198]. Furthermore, Mullooly et al. demonstrated that ADAM10 knockdown by siRNA or GI254023X reduced the migration and invasion of BT-20, MDA-MB-231, and MDA-MB-453 breast cancer cells [191]. Corroborating this finding, Tsang et al. demonstrated that ADAM10 knockdown by siRNA inhibited the proliferation and migration of MDA-MB-231 breast cancer cells [199]. Overall, these findings indicate that ADAM10 supports breast cancer cell survival via cell death inhibition and chemoresistance while supporting breast cancer cell progression via the promotion of proliferation, migration, and invasion.

#### 3.2.6. Matrix Metalloproteinase 1 (MMP1)

Among the several existing MMPs, MMP1 has been identified in EVs associated with breast cancer [93,94]. MMP1 is also overexpressed in breast cancer [200,201]. Knockdown of MMP-1 by shRNA was reported to significantly inhibit proliferation, migration, and invasion while increasing the apoptosis of MCF-7 and MDA-MB-231 breast cancer cells [200]. Moreover, an earlier study by Liu et al. demonstrated that the silencing of MMP1 inhibits the invasiveness of brain metastatic variants of MDA-MB 231 breast cancer and reduces the brain and lung metastases of breast cancer in nude mice [202]. Also, Xin et al. demonstrated that MMP1 overexpression promotes bone metastases of MCF-7 and MDA-MB-435 breast cancer in nude mice [203]. Furthermore, MMP1 upregulation was reported to potentiate tamoxifen (TAM) resistance in TAM-resistant MCF-7 breast cancer cells and increase tumor growth of TAM-resistant MCF-7 breast cancer in a xenograft mouse model [204]. Additionally, MMP1 upregulation was reported to enhance multi-drug resistance to MCF-7 breast cancer cells, as evidenced by the increase of the IC50 values of ADR, vincristine, and paclitaxel in MCF-7 breast cancer cells overexpressing MMP1 [205]. Together, these findings indicate that MMP1 contributes to breast cancer cell survival via chemoresistance while contributing to breast cancer cell progression via the promotion of invasion and metastasis.

#### 3.2.7. Peroxiredoxin (PRDX) 1 and 2

Among the PRDX isoforms, PRDX1 and PRDX2 have been identified in EVs associated with breast cancer [95]. Also, PRDX 1 and PRDX2 are highly expressed in breast cancer [206,207]. Knockdown of PRDX 1 was reported to potentiate doxorubicin (DOX)-induced apoptosis of MCF-7 [208] and MDA-MB-231 breast cancer cells [209]. Another study demonstrated that PRDX1 knockdown results in oxidative stress-induced suppression of ERα in T-47D and ZR-75-1 breast cancer cells [210] and sensitizes MCF-7, ZR-75-1, T-47D, MDA-MB-231, HCC-1806, and SK-BR-3 breast cancer cells to the cytotoxic effects of oxidative stress-inducing agents [206,211,212]. Similarly, PRDX2 knockdown sensitizes the lung metastatic variant of MDA-MB-435 breast cancer cells to oxidative stress [213] and sensitizes MCF-7 breast cancer cells to DOX-induced apoptosis [208] and ionizing radiation-induced cytotoxicity [214]. Overall, these studies suggest that PRDX1 and PRDX2 promote breast cancer cell survival via induction of radio/chemoresistance and inhibition of oxidative stress-induced cell death. Future studies are required to understand the relevance of both isoforms of the enzyme to breast cancer cell progression.

#### 3.2.8. Sirtuin 1 and 6 (SIRT1 and SIRT6)

Among the sirtuins, SIRT1 and SIRT6 have been identified in EVs associated with breast cancer [84]. Also, in breast cancer, there is an overexpression of SIRT1 [215] and SIRT6 [216]. In our recent review, we discussed the dual roles of both SIRT1 and SIRT6 in breast cancer, highlighting the tumor-suppressing and tumor-promoting effects of SIRT1 and SIRT6 in breast cancer [44]. Given the dual roles of both SIRT1 and SIRT6 in breast cancer, functional studies involving the introduction of EVs expressing SIRT1 and SIRT6 to breast cancer cells are required to determine the definitive roles of both enzymes in breast cancer. Notwithstanding, recent studies have supported the breast cancer-promoting effects of SIRT1 and SIRT6. For instance, Sahoo et al. demonstrated that SIRT1 inhibition by sirtinol, CHIC-35, or EX527 decreases the proliferation and migration of MCF-7 breast cancer cells [217], thus suggesting SIRT1 as a cell progression factor in breast cancer. The authors further demonstrated that the knockdown of SIRT1 expression decreases the viability and colony formation of MCF-7 breast cancer cells [217], thus suggesting SIRT1 as a cell survival factor in breast cancer. Also, Andreani et al. demonstrated that SIRT6 overexpression promotes migration, invasion, and lung metastasis in the Delta16HER2 mice model of breast cancer [218], thus suggesting SIRT6 as a cell progression factor in breast cancer. Although the authors further demonstrated that SIRT6 overexpression protects Delta16HER2 breast tumor cells against oxidative DNA damage [218], the contributions of the enzyme to specific breast cancer cell survival mechanisms were not investigated. Thus, more studies are required to validate the current findings and address the research gaps before a conclusion can be reached.

#### 3.2.9. Ras Homolog A and C (RhoA and RhoC) GTPases

RhoA and RhoC GTPases have been identified in EVs associated with breast cancer [96]. Consistent with this, upregulated expressions of RhoA and RhoC have been reported in breast cancer [219,220,221]. Knockdown of both RhoA and RhoC GTPases by siRNA was reported to inhibit the proliferation and invasion of MDA-MB-231 breast cancer cells [222]. Similarly, RhoA and RhoC knockdown by siRNA was also reported to reduce invasion, motility, and monolayer growth rate in SUM149 and MDA-MB-231 breast cancer cells [223]. Also, Xu et al. demonstrated that RhoC knockdown by siRNA inhibits viability, migration, and invasion but increases apoptosis and induces cell cycle arrest of SUM149 and SUM190 breast cancer cells [224]. The authors further demonstrated that the inhibition of the proliferation and invasion of SUM149 and SUM190 breast cancer cells by RhoC knockdown was via the increase of KAI1 (a tumor metastasis suppressor) and decrease of CXCR4 (a tumor progression promoter) and MMP9 [224]. Conversely, a recent study by Kalpana et al. demonstrated that RhoA expression suppresses the invasion of BT-20 and MDA-MB-231 breast cancer cells and reduces the lung and lymph node metastasis of breast cancer in mice [225]. The authors further demonstrated that RhoA suppresses invasion by reducing the expression of the tumor progression chemokines CCR5 and CXCR4 [225]. The opposing effects of RhoA GTPase as compared to RhoC GTPases may indicate the independence of both GTPases in breast cancer progression, which needs to be investigated in future studies. Also, while it is established that RhoC is relevant to breast cancer survival (via cell death inhibition) and progression (via the promotion of proliferation, invasion, and metastasis), data accumulated so far only reveals the relevance of RhoA to breast cancer cell progression. Thus, future studies are required to investigate the role of RhoA GTPase in breast cancer cell survival.

#### 3.2.10. Indoleamine-2,3-Dioxygenase (IDO)

IDO has been identified in EVs associated with breast cancer [97]. It is also consistently overexpressed in breast cancer [226,227,228,229]. Inhibition of IDO by epacadostat has been reported to induce apoptosis of MDA-MB-231 breast cancer cells [227]. Also, IDO knockdown by siRNA sensitized MCF-7 breast cancer cells to the cytotoxic effect of TAM via downregulation of IL-6. IL-6 is a TME cytokine involved in cancer chemoresistance [230]. Moreover, the upregulated expression of IDO was found to be correlated with chemoresistance in breast cancer [231]. There is an indication that IDO interacts with immune cells to promote immune evasion of breast cancer [232]. For instance, correlational studies involving patients with breast cancer have revealed that the upregulation of IDO expression is positively correlated with the increase of infiltrated regulatory T cells in situ [233]. These findings suggest that IDO is a cell survival factor in breast cancer via chemoresistance, immune cell suppression, and cell death inhibition. Furthermore, in BALB/c mice inoculated with 4T1/IDO1+ breast cancer cells, an increase in tumor growth and lung metastases was reported [234]. Also, 4T1/IDO1+ breast cancer cells showed an increased proliferation in BALB/c mice compared to 4T1/IDO1− breast cancer cells [234]. This indicates that IDO is an important factor in breast cancer cell progression. Given the limited information, studies using human breast cancer cells are required to validate the tumor progressive role of IDO in breast cancer.

#### 3.2.11. Tissue Transglutaminase (TTG)

TTG has been detected in EVs associated with breast cancer [98], while overexpressed in breast cancer [235,236]. Downregulation of TTG expression was found to sensitize MDA-MB-231 and MCF-7 breast cancer cells to DOX [237,238] and MCF-7 to ADR [239]. In addition, TTG downregulation was found to induce apoptosis of MCF-7 breast cancer cells [239]. Corroborating this finding, a recent study demonstrated that a novel TTG inhibitor, AA9, induces apoptosis of MDA-MB-231 and MCF-7 breast cancer cells [240]. Furthermore, Mangala et al. demonstrated that breast cancer cells with induced (MDA-MB-231 cells) and constitutive (MDA231/cl.16 cells) high expression of TTG are characterized by increased migrative and invasive properties [241]. Corroborating this finding, He et al. demonstrated that inhibition of TTG by shRNA results in the downregulation of EMT via an increase of E-cadherin expression and a decrease of vimentin expression in MDA-MB-231 breast cancer cells. Substantiating this finding, Seyoung et al. demonstrated that the GTP binding activity of TTG expression induces EMT via downregulation of miRNA-205 to promote bone metastasis of MCF-7 breast cancer cells in mice [242]. Moreover, using weakly migratory MDA-MB-231 breast cancer cells, Schwager et al. demonstrated that TTG knockdown by shRNA reduces metastasis, while TTG overexpression increases metastasis [243]. From the above findings, it is apparent that TTG supports breast cancer cell survival via chemoresistance and cell death inhibition while supporting breast cancer cell progression via the promotion of migration, invasion, and metastasis.

### 3.3. Enzymes Secreted in EVs Associated with Prostate Cancer (with No Current Evidence in Breast Cancer)

#### 3.3.1. Src Kinase (SK)

EVs associated with prostate cancer have been found to contain SK [100]. Consistent with this, an upregulated expression of SK has also been noted in prostate cancer [244,245]. Irwin et al. found that SK loss in transgenic adenocarcinoma of the mouse prostate (TRAMP) mice results in reduced microscopic metastasis of prostate cancer to the lung, liver, and pelvic lymph nodes [246]. This may occur through SK synergizing with androgen receptor (AR) of the prostate via upregulation of MMP9 and induction of EMT to contribute to the induction of invasive adenocarcinoma [247]. Moreover, MMP9 activity has been detected in EVs from the NB26 tumorigenic prostate cell line created by chemical mutagenesis [248], although this needs to be validated by determining the activity in EVs secreted by cells originating from actual prostate tumors. Furthermore, it has been previously established that SK potentiates androgen receptor transactivation and invasion of C4-2 prostate cancer cells [249,250]. Goc et al. also demonstrated that SK promotes growth, proliferation, and metastasis of prostate cancer in vitro (PC-3 cells) and in vivo (TRAMP mice) via activation of glycogen synthase 3 (GS3) [251]. GS3 has been implicated in several cellular functions, including energy metabolism, insulin and growth factor signaling, apoptosis, and cell division [252]. Corroborating these findings, studies investigating the effect of SK inhibition have confirmed the pivotal role of this enzyme in the promotion of prostate cancer cell progression [253,254]. Together, the above studies suggest SK as a cell progression factor in prostate cancer via the promotion of proliferation and metastasis.

Furthermore, SK has also been implicated in the survival of prostate cancer. For instance, Wu et al. demonstrated that inhibition of SK by saracatinib induces autophagy and apoptosis of PC-3 and LNCaP prostate cancer cells [255]. Additionally, the authors demonstrated that the inhibition of autophagy by 3-Methyladenine and chloroquine enhances SK inhibitor-induced apoptosis of PC-3 prostate cancer cells [255]. Findings from the above study suggest SK is an important cell survival factor of prostate cancer via cell death inhibition. Notwithstanding, more studies are required to validate the survival functions of SK before a conclusion can be reached.

#### 3.3.2. AKT1 Kinase

EVs associated with prostate cancer have been found to contain AKT1 [102]. Also, AKT1 is overexpressed in prostate cancer [256,257]. Graff et al. demonstrated that AKT1 expression is elevated in bone and soft tissue metastasis of prostate cancer compared to normal prostate tissues [257], thus indicating a role for AKT1 in the progression of prostate cancer. Supporting this, Goc et al. demonstrated that TRAMP cells with constitutive expression of AKT1 exhibit an increased migration, while TRAMP cells with dominant-negative AKT1 expression exhibit a reduced migration [258]. As a mechanism, the authors demonstrated that AKT1 activation promotes migration of PC-3 prostate cancer cells via activation of integrin [258]. Integrin αvβ3 is a cell surface receptor that participates in tumorigenesis, angiogenesis, EMT, stemness, bone metastasis, immune escape, drug resistance, and metabolic reprogramming [259]. Given the paucity of information, more studies are required to determine the mechanistic role of AKT1 in the progression and survival of prostate cancer, particularly considering the increased rate of AKT1 gene mutation in prostate cancer [260].

#### 3.3.3. TMPRSS2 Serine Protease

TMPRSS2 has been identified in EVs associated with prostate cancer [103,104,105]. Consistently, TMPRSS2 is upregulated in prostate cancer [261,262,263]. Lucas et al. demonstrated that high expression of TMPRSS2 in metastatic prostate cancer is induced by androgens [264]. The authors further showed that TMPRSS2 promotes invasion and metastasis of prostate cancer in TRAMP mice [264]. Similarly, Chun-Jung et al. demonstrated that silencing of TMPRSS2 by shRNA significantly halted dihydrotestosterone-induced invasion of LNCaP prostate cancer cells [265]. The authors further demonstrated that TMPRSS2 promotes the growth of LNCaP prostate cancer in nude mice and that TMPRSS2 promotes invasion of LNCaP prostate cancer via proteolytic activation of matriptase [265]. Matriptase is a serine protease that promotes prostate cancer invasion [266]. Moreover, it has been previously reported that TMPRSS2 proteolytic activity is crucial for prostate cancer metastasis [267]. To corroborate these findings, a recent study by Chun-Jung et al. demonstrated that overexpression of TMPRSS2 significantly increases the invasion of LNCaP, PC-3, and DU145 prostate cancer cells [268]. The authors further demonstrated that TMPRSS2 inhibition by hepatocyte growth factor activator inhibitor-2 abrogates the TMPRSS2-induced invasion of the prostate cancer cells and that activated matriptase levels correlate with TMPRSS2 expression in LNCaP and VCaP prostate cancer cells [268]. Together, these studies indicate that TMPRSS2 proteolytically activates its substrates (such as matriptase) to promote prostate cancer progression via an increase in tumor growth, invasion, and metastasis. Future studies are required to investigate the contributions of TMPRSS2 to the survival of prostate cancer.

#### 3.3.4. Transglutaminase-4 (TGM4)

EVs associated with prostate cancer have been found to express TGM4 [104]. Consistent with this, TGM4 expression is upregulated in prostate cancer [269,270]. In contrast, downregulated expressions of TGM4 have also been reported in certain prostate cancer cells [270]. This may suggest a wide range of TGM4 expressions in prostate cancer. Knockdown of TGM4 by ribozyme transgene reduced adhesion, motility, and invasiveness of CAHPV-10 prostate cancer cells, while forced expression of TGM4 increased adhesion, motility, and invasion of PC-3 prostate cancer cells [270,271,272]. Moreover, TGM4 overexpression in PC-3 prostate cancer cells reversed the adhesion, growth, and migration inhibitory effect of melanoma differentiation-associated gene-7/interleukin-24 (MDA-7/IL-24) [273]. MDA-7/IL-24 is a multifunctional cancer-killing cytokine [274]. Corroborating these findings, TGM4 overexpression increased migration of PC-3, PZ-HPV-7, and DU145 prostate cancer cells, while TGM4 knockdown by ribozyme transgene reduced EMT of CAHPV-10 prostate cancer cells [275]. Together, these findings suggest TGM4 as a cell progression factor in prostate cancer via the promotion of adhesion, growth, migration, invasion, and EMT. However, the role of TGM4 in the survival of prostate cancer needs to be investigated, particularly considering its opposing effect on MDA-7/IL-24.

#### 3.3.5. Six-Transmembrane Epithelial Antigen of Prostate (STEAP) 1 and 2

Among the STEAP isoforms, STEAP1 and STEAP2 have been identified in EVs associated with prostate cancer [104,107]. Consistent with this, the two STEAP isoforms are overexpressed in prostate cancer [276,277,278]. There is limited information on the relevance of STEAP1 and STEAP2 to the progression and survival of prostate cancer. Notwithstanding, Sandra et al. demonstrated that knockdown of STEAP1 by siRNA reduces viability and proliferation and downregulates survival pathways (such as AKT and ERK signaling) of LNCaP and C4-2B prostate cancer cells [279]. Surprisingly, the authors further demonstrated that STEAP1 knockdown by siRNA reversed the sensitivity of LNCap prostate cancer cells to the apoptotic effects of paclitaxel and cabazitaxel [279]. These findings suggest STEAP1 is an important survival factor in prostate cancer via chemoresistance and cell death inhibition. Future studies are required to investigate the contribution of STEAP1 to the progression of prostate cancer. On the other hand, Jones et al. demonstrated that STEAP2 knockdown by CRISPR/Cas9 reduces proliferation, migration, and invasion of LNCaP and C4-2B prostate cancer cells [280]. Corroborating this finding, Burnell et al. demonstrated that STEAP2 knockdown by siRNA reduces proliferation, migration, and invasion of PC-3 and LNCaP prostate cancer cells [281]. While there are no studies on the relevance of STEAP2 to prostate cancer survival, the above studies suggest STEAP2 as a cell progression factor in prostate cancer via the promotion of proliferation, migration, and invasion.

#### 3.3.6. Hyaluronidase 1 (HYAL1)

Among the HYAL isoforms, HYAL1 has been identified in EVs associated with prostate cancer [108,109]. Also, HYAL1 has been reported to be overexpressed in prostate cancer [282]. Demonstrating the relevance of HYAL1 to the progression of prostate cancer, Kovar et al. demonstrated that overexpression of HYAL1 induced lymph node metastasis in an orthotopic mouse model of prostate cancer [283]. Additionally, McAtee et al. demonstrated that overexpression of HYAL1 increased the proliferation and motility of 22Rv1 prostate cancer cells [109]. Together, these studies suggest HYAL1 as a cell progression factor in prostate cancer via the promotion of proliferation, motility, and metastasis. Notwithstanding, future studies are required to validate the current findings and investigate the relevance of HYAL1 to prostate cancer cell survival before a conclusion can be reached on its mechanistic relevance.

## 4. Clinical Potential of EVs in Breast and Prostate Cancers

**Figure 4:**
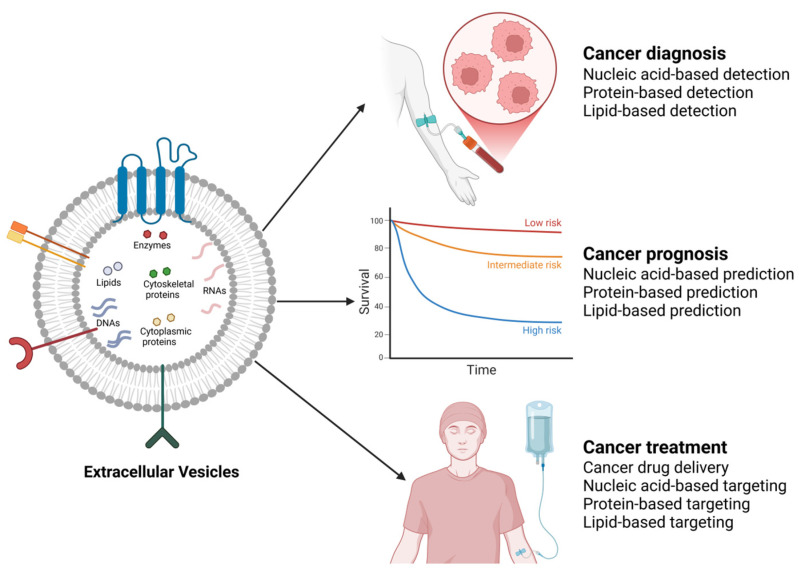
Clinical potential of cancer-associated EVs. By harnessing EVs through various approaches guided by their cargo (nucleic acid-based, protein-based, and lipid-based approaches), the diagnosis, prognosis, and treatment of various cancers, including breast and prostate cancers, could be achieved. The image was created with BioRender.com (accessed on 17 February 2025).

EVs have been shown to induce the production of factors that create a favorable microenvironment for tumorigenesis [284]. As a result, research is now focused on investigating the potential of EVs and their molecules in the diagnosis, prognosis, and treatment of cancer (Figure 4). While research on the clinical potential of EVs in breast and prostate cancers is ongoing [285,286,287], it is pertinent to highlight some promising outcomes of existing research in the field. For instance, in breast cancer treatment, it has been demonstrated in vitro that EVs could serve as delivery vessels for various anticancer drugs, such as DOX and ADR, to improve targeting efficiency and reduce side effects [288]. Likely, this approach could also be effective in the treatment of prostate cancer.

Additionally, as demonstrated in a study using EVs from mesenchymal stem cells to transfer miR-145 into T-47D breast cancer cells to inhibit T-47D breast cancer metastasis [289], functional small RNAs (e.g., siRNAs and miRNAs) can be packaged into EVs to downregulate target genes involved in the progression of both breast and prostate cancers. Furthermore, in a study by Huang et al., engineered EVs were used as in situ dendritic cells-primed vaccine to boost antitumor immunity in mice with orthotopic TNBC [290], thus suggesting the potential of engineered EVs in the treatment of breast cancer. The use of engineered EVs with antitumor functions could also be explored in the treatment of prostate cancer.

Given the potential of tumor-derived EVs in the discovery of novel cancer biomarkers, studies have explored the potential of EV molecules in the diagnosis and prognosis of breast cancer [291]. Perhaps one of the most significant breakthroughs in this area of research is the development of a microfluidic chip-based EV mRNA sensor for the detection of HER2-positive breast cancer via quantitation of EV ERBB2 in the blood [292]. Other promising EV biomarkers for breast cancer include miR-200a, miR-200c, and miR-205 [293]. In the diagnosis and prognosis of prostate cancer, miRNA-196a-5p and miRNA-501-3p in EVs isolated from urine were found to be diagnostic markers for patients with advanced prostate cancer [294], while miRNA-1290 and miRNA-375 were associated with poor prognosis of patients with castrate-resistant prostate cancer [295]. Other promising EV biomarkers for prostate cancer include miR-107, miR-375, and PCA-3 [293].

Notwithstanding, to fully explore the clinical potential of EVs in the diagnosis, prognosis, and treatment of breast and prostate cancers, future studies are required to identify, quantitate, and functionally analyze cell survival and progression molecules in EVs secreted by breast and prostate cancers. For instance, the expression pattern of functionally relevant EV molecules (such as enzymes) needs to be determined in EVs derived from breast and prostate cancers to ascertain their usefulness in the early diagnosis of both cancers [296]. Most importantly, breast and prostate cancer-derived EVs with high or low expression of enzymes can be incorporated into breast and prostate cells, respectively, to determine their contribution to cell survival and progression. This will provide clues for the development of therapeutic strategies against both cancers. Specifically, the enzyme cargo of EVs from breast and prostate cancers could be targeted as part of the therapy against both cancers by (1) modifying the enzyme(s) in the EVs, (2) preventing its release into both breast and prostate cancer cells, and (3) interfering with its contributions to survival and progression of both cancers via targeted inhibition of the enzyme(s). However, a major setback to the clinical application of EVs is the lack of standardized methods for EV isolation, characterization, and downstream analyses [297]. This has resulted in potential contamination of EV particles by non-EV particles, thus negatively affecting the reproducibility, reliability, and clinical translation of findings from EV research [298]. To circumvent this challenge, transparent reporting, interlaboratory comparison studies, and standardization of pre-analytical procedures have been recommended, among other strategies for improving the reliability and clinical translation of EV research [298].

## 5. Conclusions

EVs provide valuable clues to unraveling the pathogenesis of various cancers. In their cargoes, EVs include both catalytic (e.g., enzymes) and non-catalytic (e.g., non-catalytic proteins and nucleic acids) molecules that create a microenvironment for tumorigenesis. Several enzymes that are constitutively upregulated in breast and prostate cancers have been identified in EVs secreted by both cancers. In this review, we discussed the functional relevance of enzymes in EVs associated with breast and prostate cancers, emphasizing their relevance to the progression and survival of both cancers. Among the enzymes discussed, ACLY, ENO, FASN, FAK, and PK are found in EVs from both breast and prostate cancers and contribute to the progression and survival of both cancers. Key mechanisms of cell progression influenced by these enzymes are adhesion, proliferation, migration, invasion, and metastasis, whereas mechanisms of cell survival influenced are chemoresistance, radioresistance, angiogenesis, cell death inhibition, cell colony formation, and immune evasion. Given this information, future studies are required to validate the current findings, address the research gaps, and explore the usefulness of these EV enzymes in the diagnosis, prognosis, and treatment of breast and prostate cancers.

## Figures and Tables

**Table 1:** Malignant functions of enzymes secreted in EVs associated with breast cancer.

Enzyme	Detection/Quantitation Method	Biological Function	Source of EVs	Malignant Functions	**References**
ATP citrate lyase	Protein-based (LC-MS/MS analysis, Western blotting, and ACLY activity assay)	Converts citrate to acetyl-CoA and oxaloacetate in the presence of coenzyme A and ATP	MCF-7 and MDA-MB-231 breast cancer cells	Promotes survival of breast cancer	[84]
Enolase 1 and 2	Protein-based (LC-MS/MS analysis and Western blotting)	Converts 2-phosphoglycerate (2-PG) to phosphoenolpyruvate (PEP)	MCF-7, MDA-MB-231, and T-47D breast cancer cells	Promotes progression and survival of breast cancer	[85,86,87]
Fatty acid synthase	Protein-based (LC-MS/MS analysis and Western blotting)	Synthesizes the fatty acid palmitate from acetyl-CoA and malonyl-CoA in the presence of NADPH	MCF-7 breast cancer cells	Promotes progression and survival of breast cancer	[87]
Focal adhesion kinase	Protein-based (Western blotting)	Phosphorylates the OH group of tyrosine amino acid residue of specific protein targets	Peripheral blood from patients with breast cancer	Promotes progression and survival of breast cancer	[88]
Pyruvate kinase M2	Protein-based (LC-MS/MS analysis and Western blotting) and mRNA-based (Double-tagging RT-PCR)	Transfers a phosphate group from phosphoenolpyruvate (PEP) to adenosine diphosphate (ADP), producing pyruvate and ATP	MCF-7 and MDA-MB-231 breast cancer cells	Promotes progression and survival of breast cancer	[86,87]
Phosphoglycerate kinase 1	mRNA-based (Double-tagging RT-PCR)	Reversibly transfers a phosphate group from 1,3-bisphosphoglycerate (1,3-BPG) to ADP, producing 3-phosphoglycerate (3-PG) and ATP	MCF-7 and MDA-MB-231 breast cancer cells	Promotes progression and survival of breast cancer	[86]
Phosphoglycerate mutase 1	mRNA-based (Double-tagging RT-PCR)	Converts 3-phosphoglycerate (3PG) to 2-phosphoglycerate (2PG) through a 2,3-bisphosphoglycerate intermediate	MCF-7 and MDA-MB-231 breast cancer cells	Promotes progression and survival of breast cancer	[86]
Glucose-6-phosphate dehydrogenase	Protein-based (LC-MS/MS analysis and Western blotting)	Converts glucose 6-phosphate in the presence of nicotinamide adenine dinucleotide phosphate (oxidized) to 6-phospho-D-glucono-1,5-lactone, producing NADPH	MCF-7 breast cancer cells	Promotes cell survival of breast cancer	[87]
Glyceraldehyde 3-phosphate dehydrogenase	Protein-based (LC-MS/MS analysis and Western blotting) and mRNA-based (Double-tagging RT-PCR)	Converts glyceraldehyde 3-phosphate to D-glycerate 1,3-bisphosphate	MCF-7 and MDA-MB-231 breast cancer cells; plasma from patients with breast cancer	Promotes survival of breast cancer	[86,87,89]
A Disintegrin and Metalloproteinase 9 and 10	Protein-based (LC-MS/MS analysis and Western blotting)	Cleaves extracellular portions of transmembrane proteins to release soluble ectodomains from the cell surface	Hs578T, BT-549, MDA-MB-231, LM2 breast cancer cells; plasma of patients with breast cancer	Promotes progression and survival of breast cancer	[90,91,92]
Matrix metalloproteinase 1	Protein-based (LC-MS/MS analysis and Western blotting)	Degrades extracellular matrix proteins, such as gelatin and collagen IV and V.	MDA-MB-231-HM breast cancer cells; urine of patients with breast cancer	Promotes progression of breast cancer	[93,94]
Peroxiredoxin 1 and 2	Protein-based (LC-MS/MS analysis and Western blotting)	Reduces hydrogen peroxides and different alkyl hydroperoxides to water	MDA-MB-231 breast cancer cells	Promotes progression and survival of breast cancer	[95]
Sirtuin 1 and 6	Protein-based (LC-MS/MS analysis, Western blotting, and SIRT1 and SIRT6 activity assay)	Deacetylates protein targets and/or adds ADP-ribose moieties to protein targets	MCF-7 and MDA-MB-231 breast cancer cells	Promotes progression of breast cancer	[84]
RhoA/RhoC GTPase	Protein-based (Western blotting)	Binds and hydrolyses nucleotide guanosine triphosphate to guanosine diphosphate	MDA-MB-231, SK-BR-3, and HCC70 breast cancer cells	Promotes progression and survival of breast cancer	[96]
Indoleamine-2,3-dioxygenase	Protein-based (Western blotting) and mRNA-based (RT-PCR)	Oxidizes L-tryptophan to form N-formylkynurenine	Plasma from patients with breast cancer	Promotes progression and survival of breast cancer	[97]
Tissue transglutaminase	Protein-based (Western blotting and transamidation activity)	Crosslinks proteins between an ε-amino group of lysine and a γ-carboxamide group of glutamine, forming a proteolytically resistant inter- or intramolecular bond	MDA-MB-231 breast cancer cells	Promotes survival of breast cancer	[98]

**Table 2:** Malignant functions of enzymes secreted in EVs associated with prostate cancer.

Enzyme	Detection/Quantitation Method	Biological Function	Source of EVs	Malignant Functions	**References**
ATP citrate lyase	Protein-based (Western blotting and LC-QTOF MS analysis)	Converts citrate to acetyl-CoA and oxaloacetate in the presence of coenzyme A and ATP	DU145, VCaP, and LNCaP prostate cancer cells	Promotes survival of prostate cancer	[99]
Enolase 1 and 2	Protein-based (Western blotting and LC-QTOF MS analysis)	Converts 2-phosphoglycerate (2-PG) to phosphoenolpyruvate (PEP)	DU145 and VCaP prostate cancer cells	Promotes progression and survival of prostate cancer	[99]
Fatty acid synthase	Protein-based (Western blotting and LC-QTOF MS analysis)	Converts acetyl-CoA and malonyl-CoA to a 16-carbon fatty acid palmitate	DU145, VCaP, LNCaP, and C4–2 prostate cancer cells	Promotes progression and survival of prostate cancer	[99]
Focal adhesion kinase	Protein-based (Western blotting)	Phosphorylates the OH group of tyrosine amino acid residue of protein targets	PC-3 prostate cancer cells	Promotes progression and survival of prostate cancer	[100]
Pyruvate kinase M2	Protein-based (Western blotting and LC-QTOF MS analysis)	Converts phosphoenolpyruvate and ADP into pyruvate and ATP	VCaP, C4–2, LNCaP, DU145, and PC-3 prostate cancer cells	Promotes progression and survival of prostate cancer	[99,101]
Src kinase	Protein-based (Western blotting)	Phosphorylates the OH group of tyrosine amino acid residue of specific protein targets	C4-2B, PC-3, and DU145 prostate cancer cells	Promotes progression and survival of prostate cancer	[100]
Akt1 kinase	Protein-based (Western blotting and Akt kinase activity assay)	Phosphorylates the OH group of serine or threonine amino acid residue of protein targets	Plasma of patients with metastatic prostate cancer	Promotes progression of prostate cancer	[102]
TMPRSS2 serine protease	Protein-based (Western blotting and Akt Kinase activity assay) and mRNA-based (RT-PCR)	Cleaves peptide bonds in protein targets	PC-3 and LNCaP prostate cancer cells; and Urine/Plasma of patients with prostate cancer	Promotes progression of prostate cancer	[103,104,105]
Transglutaminase-4	Protein-based (Western blotting) and mRNA-based (RT-PCR)	Forms an isopeptide bond between γ-carboxamide groups (-(C=O)NH_2_) of glutamine residue side chains and the ε-amino groups (-NH_2_) of lysine residue side chains	Urine of patients with prostate cancer	Promotes progression of prostate cancer	[104,105,106]
STEAP1 and 2	Protein-based (Western blotting) and mRNA-based (RT-PCR)	Catalyzes the reduction of Iron and Copper	Plasma and Urine of patients with prostate cancer	Promotes progression and survival of prostate cancer	[104,107]
Hyaluronidase 1	Protein-based (Western blotting)	Cleaves β1→4-N-acetylglucosaminide bonds of intracellular hyaluronan of all sizes	22Rv1 human prostate adenocarcinoma cells	Promotes progression of prostate cancer	[108,109]

## Data Availability

Not applicable.
